# Hierarchical SAPO-34 Catalysts as Host for Cu Active Sites

**DOI:** 10.3390/ma16165694

**Published:** 2023-08-19

**Authors:** Julio C. Fernandes Pape Brito, Ivana Miletto, Leonardo Marchese, Daniel Ali, Muhammad Mohsin Azim, Karina Mathisen, Enrica Gianotti

**Affiliations:** 1 Department for Sustainable Development and Ecological Transition, Università del Piemonte Orientale, Piazza Sant’Eusebio 5, 13100 Vercelli, Italy; 2 Department of Pharmaceutical Sciences, Università del Piemonte Orientale, Largo Donegani 2, 28100 Novara, Italy; 3 Department of Science and Technological Innovation, Università del Piemonte Orientale, Via T. Michel 11, 15100 Alessandria, Italy; leonardo.marchese@uniupo.it; 4 Department of Chemistry, Norwegian University of Science and Technology (NTNU), 7491 Trondheim, Norway

**Keywords:** hierarchical SAPO-34, Cu active sites, Cu speciation, heterogeneous catalysis, propene or isobutene as reductant, SCR of NO, UV-Vis spectroscopy

## Abstract

Cu-containing hierarchical SAPO-34 catalysts were synthesized by the bottom-up method using different mesoporogen templates: CTAB encapsulated within ordered mesoporous silica nanoparticles (MSNs) and sucrose. A high fraction of the Cu centers exchanged in the hierarchical SAPO-34 architecture with high mesopore surface area and volume was achieved when CTAB was embedded within ordered mesoporous silica nanoparticles. Physicochemical characterization was performed by using structural and spectroscopic techniques to elucidate the properties of hierarchical SAPO-34 before and after Cu introduction. The speciation of the Cu sites, investigated by DR UV-Vis, and the results of the catalytic tests indicated that the synergy between the textural properties of the hierarchical SAPO-34 framework, the high Cu loading, and the coordination and localization of the Cu sites in the hierarchical architecture is the key point to obtaining good preliminary results in the NO selective catalytic reduction with hydrocarbons (HC-SCR).

## 1. Introduction

One of the greatest challenges of our age is climate change mitigation and, in this context, the reduction of nitrogen oxides (NO, NO_2_, N_2_O, referred to as NO_x_) in the atmosphere represents one of the most urgent needs. In fact, NO_x_, which is emitted from power stations, factories and vehicular traffic, is now considered to be one of the major sources of atmospheric pollutants and significantly influences the global tropospheric chemistry and our wellness. In the past few years, a research topic that has become more and more popular deals with transition and noble metals (e.g., Cu, Fe, Ce, Mn, Ag, Pt) exchanged into microporous zeolites (ZSM-5, Beta zeolite) [1,2]. In particular, there is a raised interest in Cu-containing zeolites as catalysts for the abatement of NO_x_ through the selective catalytic reduction (SCR) process, which is an effective technology to remove hazardous NO_x_. Among the various zeolitic systems, silicoaluminophosphate (SAPO), microporous crystalline zeo-type materials are widely used as heterogeneous catalysts because of their moderated acidity and well-defined microporous structure. In particular, SAPO-34, with a chabazite (CHA) topological structure and 8-ring pore opening, represents a good heterogeneous catalyst in terms of intermediate and product shape selectivity in important industrial reactions such as methanol to light olefins (MTO) processes [3,4,5].

SAPO-34 microporous architecture is also suitable to host Cu sites in a well-defined position, associated with charge compensating AlO_4_^−^ tetrahedra sites that are distributed throughout the microporous framework, creating in this way the active sites to perform selective catalytic reduction (SCR) of NO_x_ [6,7,8]. However, the narrow pore aperture (3.8 × 3.8 Å) of the CHA structure severely affects the substrate diffusion in the micropores and enhances the possibility of catalyst deactivation due to coke formation and deposition within the micropores. Generally, molecular transport has been regarded as the rate-determining step of the catalytic reaction since the narrow channels of zeolitic catalysts had a strong impact on mass transfer. In recent years, many efforts have been addressed in the optimization of the framework structure to eliminate diffusion barriers in zeolite catalysts, such as reducing crystal sizes, regulating morphology by exhibiting specific crystallographic planes, and introducing mesoporous networks into microporous zeolitic architectures [9]. Particularly, the latter approach has led to the development of hierarchical porous materials that combine two levels of porosity (micro- and mesoporosity), expecting an improvement in the catalytic performances owing to the overcoming of the diffusion limitation in the micropore network [10,11]. Hierarchical zeolitic structures can be synthesized using both top-down [12,13] and bottom-up [14] approaches. The top-down approach, widely used for the production of hierarchical zeolites, is a post-synthetic modification of the microporous structure, such as demetallation under acidic or basic conditions, to extract framework constituents while preserving crystallinity. Nevertheless, the generation of mesoporous voids can induce a localized collapse of the framework and further increase the number of defect sites, thus altering both the textural and acid characteristics of the parent framework [15].

In the case of SAPO structures, it has been reported that the top-down method can irreversibly alter the microporous structure because of the relative instability of the framework under strongly acidic or alkaline conditions [12], and, for these reasons, a bottom-up approach is usually preferable. Although needful of a precise design, the aforementioned approach is characterized by its simplicity, as it is capable of being employed in a “one-pot” synthesis process. Furthermore, it provides ample opportunities for achieving a significant level of control over the structure by means of modifying the micellar arrangement through the careful selection of surfactant molecules [15]. Therefore, hierarchical SAPO-34 catalysts, characterized by an additional mesoporous network connected with the microporous structure, have been synthesized using a bottom-up approach to ensure a high degree of structural control of the SAPO architecture with the retention of the Brønsted acid sites, in contrast with the top-down method to produce hierarchical materials. In fact, the presence of Brønsted acid sites is essential owing to their exchangeable capacity that allows the introduction of metal cations [8]. Among the various metal cations that are used for catalytic purposes, copper is particularly noteworthy for its redox properties, specifically the Cu (II)/Cu(I) couple. Owing to this feature, copper has extensive applications, especially when it is introduced in a small pore zeo-type system such as the CHA architecture. In such a configuration, copper serves as the active site for the selective catalytic reduction of NO_x_ using a wide range of reductants. Furthermore, some studies have shown that the presence of additional mesoporous channels within the zeolite catalytic architecture results in a higher dispersion of the metal component in comparison to conventional microporous materials. Consequently, this enhanced dispersion ultimately leads to an enhanced catalytic activity [16]. Typically, the synthesis of hierarchical SAPO-34 through the bottom-up approach requires the presence of two structure-directing agents (SDAs), one to form the microporous structure and the other to induce the mesopore channel development.

In this article, two types of hierarchical Cu-containing SAPO-34 materials with the same Si loading were synthesized by the bottom-up approach using two different mesoporogenic templates: CTAB (hexadecyltrimethylammonium bromide) encapsulated within ordered mesoporous silica nanoparticles (MSNs) and sucrose, a simple, low-cost, and widely commercially available molecule [8,17]. In both cases, tetraethylammonium hydroxide (TEAOH) was used as a template to induce the micropore formation typical of the CHA structure. Cu cations were introduced following a single-step ion exchange strategy into as-synthesized SAPO-34, still containing SDAs (for both microporous and mesoporous network development). This method allows for reducing the number of synthetic steps as well as avoiding the drawbacks of the traditional multistep method, widely reported in the literature [8]. In addition, this synthetic approach ensures the localization of the Cu ions in the position adjacent to the 6-membered window of the CHA structure, as evidenced by diffuse reflectance UV-Vis (DR-UV-Vis) characterization, which seems fundamental for the activity in the NO_x_ abatement through a selective catalytic reduction reaction. The hierarchical Cu-exchanged catalysts were synthesized, characterized by using a structural technique (XRD), volumetric analysis (N_2_ adsorption/desorption isotherms at 77 K), and electronic spectroscopy (diffuse reflectance UV-Vis) to evidence the oxidation state and coordination of Cu-exchanged sites, and finally tested in a selective catalytic reduction of NO_x_ using different hydrocarbons as reductants (HC-SCR).

## 2. Materials and Methods

### 2.1. Synthesis of Hierarchical (HPS34CTAB, HPS34SUC) and Microporous SAPO-34 (S34) Catalysts

Hierarchical SAPO-34 catalysts were synthesized using a gel of molar composition 1.0 Al/1.0 P/0.6 Si/0.067 SDA_meso_/1 TEAOH/60 H_2_O, where the SDA_meso_ was sucrose in the case of HPS34SUC and CTAB encapsulated within ordered mesoporous silica nanoparticles (which serves also as a silicon precursor) in the case of HPS34CTAB.

*HPS34CTAB*. Aluminum isopropoxide (7.00 g, Sigma Aldrich, Milano, Italy) was added to 15 mL of deionized water while stirring, and tetraethylammonium hydroxide (TEAOH) (14.00 mL, 35 wt% in H_2_O, Sigma Aldrich, Milano, Italy) was added dropwise; the mixture was stirred at room temperature for 1 h. Ordered mesoporous silica nanoparticles with hexadecyltrimethylammonium bromide encapsulated within the mesopores (CTAB, SDA_meso_) (2.06 g, Sigma Aldrich, Milano, Italy) were suspended in 15 mL of deionized water and slowly added to the reaction mixture, which was further stirred for 2 h. Phosphoric acid (2.33 mL, 85 wt% in H_2_O, Sigma Aldrich, Milano, Italy) was added dropwise under stirring and the gel was further vigorously stirred for 30 min to produce a white gel, which was then transferred to a Teflon-lined stainless-steel autoclave and crystallized at 473 K for 60 h under autogenous pressure. The solid product from the autoclave was then recovered by filtration and washed with water.

Ordered mesoporous silica nanoparticles (MSNs) were prepared using cetyltrimethylammoniumbromide (CTAB) as a structure-directing agent. CTAB (1.9 mmol) was first dissolved in 340 mL of water. Then aqueous NaOH (2.0 M, 2.45 mL) was added to the CTAB solution, followed by adjusting the solution temperature to 80 °C. Finally, tetraethoxysilane (TEOS, 3.5 mL, 18.1 mmol) was added simultaneously dropwise to the solution for a period of 4 min. The mixture was stirred at 80 °C for 2 h to give rise to a white precipitate. The solid product was filtered, washed with deionized water and ethanol, and dried in vacuo.

*HPS34SUC*. The reaction was carried out as reported for HPS34CTAB, with the only difference being that amorphous silica fumed (1.24 g, Sigma Aldrich, Milano, Italy) was added before the addition of the microporous template (TEAOH), and sucrose previously dissolved in water (0.79 g, Sigma Aldrich, Milano, Italy) was used as SDA_meso_.

*S34*. The reaction was carried out as reported for the hierarchical analogue without the use of any SDA_meso_ and used silica fumed as the silicon precursor (1.24 g, Sigma Aldrich, Milano, Italy).

A fraction of the as-prepared products were dried in air at 373 K and directly used, without removing the templates, in the liquid aqueous solution (LIE) copper exchange procedure. The remaining aliquots were calcined in a tube furnace under airflow at 873 K for 16 h to remove organic surfactants and the micropore template, producing white crystalline solids. The calcined samples were prepared to perform the analysis on the hierarchical and microporous materials before the Cu exchange.

### 2.2. Preparation of Copper-Containing Catalysts

Hierarchical and microporous Cu-exchanged SAPO-34 catalysts were prepared by using a direct liquid aqueous solution ion exchange (LIE) method starting from as-synthesized materials containing all the templates [8]. A 0.2 M aqueous solution of CuSO_4_ 5H_2_O (Sigma Aldrich, Milano, Italy) was used for the copper exchange. The Cu ion exchange of as-synthesized SAPOs containing SDAs was carried out in the copper solution at 243 K for 4 h under vigorous stirring. The colored solids were then recovered by filtration, washed several times with water, and finally calcined in airflow at 873 K for 4 h.

### 2.3. Physico-Chemical Characterization

N_2_ physisorption measurements were carried out at 77 K in the relative pressure range from 1 × 10^−6^ to 1 P/P_0_ by using a Quantachrome (Boynton Beach, FL, USA) Autosorb1MP/TCD instrument. Prior to the analysis, the samples were outgassed at 573 K for 3 h (residual pressure range lower than 10^−6^ Torr) in order to remove water and other adsorbed species. Specific surface areas (SSAs) were determined by the Brunauer–Emmett–Teller (BET) method in the relative pressure range from 0.01 to 0.1 P/P_0_. The desorption branch was analyzed by the NLDFT (non-local density functional theory) method to obtain the micro- and mesoporous surface areas and pore size distributions of the hierarchical and microporous samples.

X-ray powder diffraction (XRPD) patterns were obtained using a Bruker AXS D8 ADVANCE diffractometer (Karlsruhe, Germany), in reflection mode with Bragg–Brentano geometry, operating with a radiation source of monochromatic X-rays Cu Kα (λ = 1.5406 Å) and an LYNXEYE_XE_T high-resolution position-sensitive detector. XRPD patterns were recorded in the 5–40° (2θ) range at the voltage and amperage of the source 40 kV/40 mA, with a coupled 2θ-θ method, at a scan speed of 0.100 s/step and a step size of 0.01°.

Diffuse reflectance (DR) UV-Vis-NIR spectra were recorded with a Perkin-Elmer Lambda 900 UV-Vis-NIR spectrophotometer (Waltham, MA, USA) equipped with the integrating sphere optical system that allows recording in diffuse reflectance mode. DR UV-Vis-NIR spectra were recorded in the spectral range 2500–200 nm (50,000–4000 cm^−1^) at 1 nm of resolution, measuring the reflectance (%R) with respect to the reference spectrum of the BaSO_4_.

### 2.4. Catalytic Tests

The hierarchical and microporous Cu-containing catalysts (30 mg) were transferred to a glass-lined steel reactor and kept in place with quartz wool. All samples were heated overnight in Ar (5 mL/min) reaching a temperature of 773 K and activated in O_2_ (2% in He) at 773 K for 1 h. The reaction was set to proceed from 773 K to 548 K with temperature intervals of 25 K using a gas reaction mixture of NO (2000 ppm), propene (5000 ppm), and 2% O_2_, giving a weight hourly space velocity of 2.35 g_reactants_/g_catalyst_ per hour. Following this, a new sample was inserted into the reactor and after the activation procedure, isobutane was introduced as the hydrocarbon reductant at the reaction temperature of 748 K, under what were otherwise the same reaction conditions. The NO conversion for each step was calculated according to Equation (1):

NO conversion [%] = ((NO_IN_ − NO_OUT_)/NO_IN_) × 100 (1)

## 3. Results and Discussion

Two different hierarchical SAPO-34 catalysts were synthesized by the bottom-up method using TEAOH as the structure-directing agent for the micropores and CTAB encapsulated within MSNs (HPS34CTAB) or sucrose (HPS34SUC) as the templates for the mesoporous network (SDA_meso_). The hierarchical materials were obtained starting from a synthetic gel of molar composition 1.0 Al/1.0 P/0.6 Si/0.067 SDA_meso_/1 TEAOH/60 H_2_O, which was reacted under hydrothermal conditions at 473 K for 72 h under autogenous pressure. For the sake of comparison, microporous SAPO-34 (S34) was prepared under identical conditions, excluding the presence of a mesoporogenic template. The morphology and the shape of the hierarchical and microporous SAPO-34 catalysts investigated using SEM microscopy were similar, as reported in ref. [8].

The textural properties of calcined hierarchical SAPO-34, before Cu exchange, were assessed by N_2_ adsorption/desorption volumetric analysis at 77 K and compared to the textural properties of the microporous SAPO-34 reference material (Table 1 and Figure S1 in the Supplementary Materials). Hierarchical SAPO-34 materials presented a Type IV isotherm with a hysteresis loop characteristic of a mesoporous system, while the Type I isotherm was observed for microporous SAPO-34, as expected. The pore size distribution of hierarchical catalysts, obtained by using the NLDFT (non-localized density functional theory) method [18,19] on the desorption branch of their respective isotherms, evidenced the presence of two families of mesopores characterized by diameters of around 35 and 50 Å. Furthermore, substantial enhancements in mesopore volume (V_meso_), total pore volume (V_tot_), and mesopore surface area (S_meso_) were detected, strongly supporting the successful preparation of hierarchical SAPO-34 characterized by the coexistence of multiple levels of porosity. In particular, hierarchical SAPO-34 prepared with CTAB encapsulated within ordered mesoporous silica nanoparticles (HPS34CTAB) showed a higher mesopore surface area, volume, and a higher fraction of mesopores with a diameter of around 35 Å with respect to the hierarchical material prepared using sucrose as mesoporogen.

Cu-exchanged hierarchical and microporous SAPO-34 were prepared by a direct liquid aqueous solution ion exchange (LIE) method at 343 K using a 0.2 M solution of CuSO_4_ 5H_2_O as the copper source, without removing the structure-directing agents [8]. In Table 2, the acronyms, the theoretical framework composition, the structure-directing agents (SDAs) used to template the microporous and the mesoporous networks together with the Cu loadings, calculated using the Lambert–Beer equation on the UV-Vis spectra of eluate after the washing procedure (Figure S2 in the Supplementary Materials) of the Cu-containing hierarchical catalysts are reported and contrasted to the data obtained for the microporous SAPO-34. It is worth noting that the highest Cu loading was observed for the Cu-containing HPS34CTAB sample.

**Figure 1:**
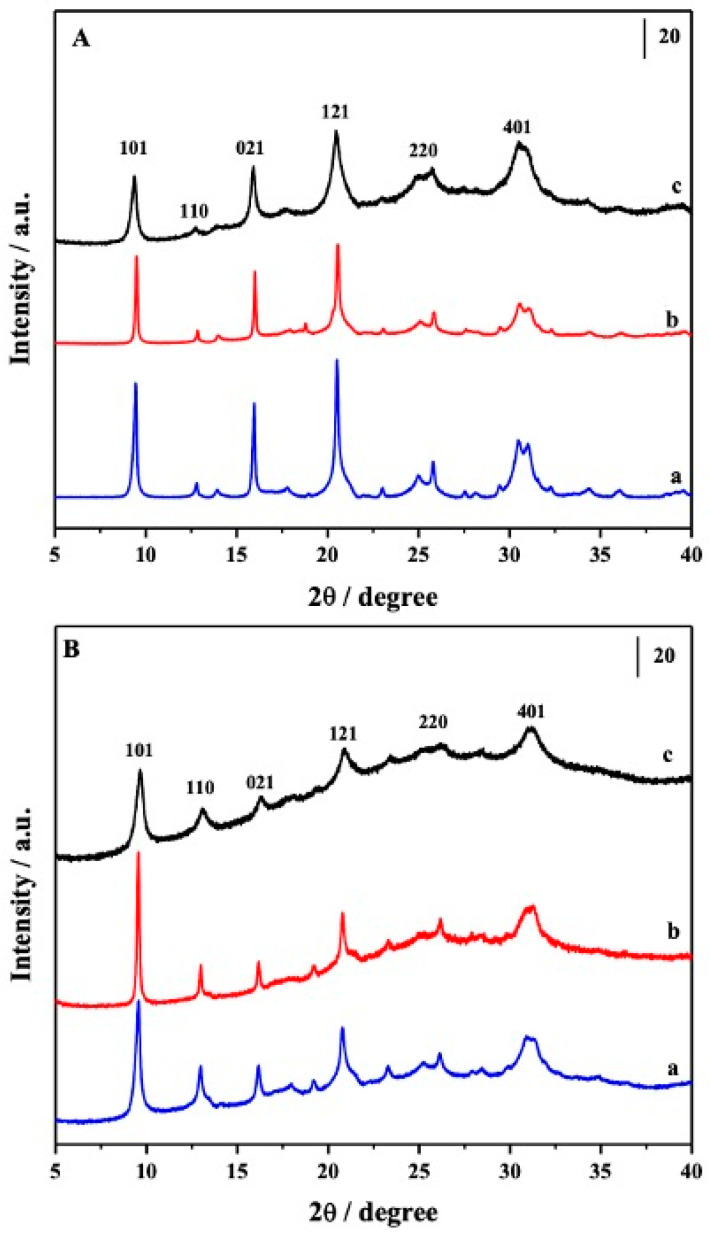
Section (**A**): XRPD pattern of HPS34SUC (curve a), HPS34CTAB (curve b), and S34 (curve c). Section (**B**): XRPD pattern of the samples after Cu exchange; Cu/HPS34SUC (curve a), Cu/HPS34CTAB (curve b), and Cu/S34 (curve c).

The structural characterization of the catalysts, performed by X-ray powder diffraction (XRPD), evidenced the diffraction pattern typical of CHA structure, confirming the phase purity and crystallinity in both hierarchical catalysts (Figure 1A). After the Cu exchange (Figure 1B), signals of CuO particles, expected at 35.6 and 38.8 2θ degrees, were not formed; nevertheless, a broad feature between 20 and 35 2θ degrees, assigned to the presence of an amorphous phase, can be observed.

**Figure 2:**
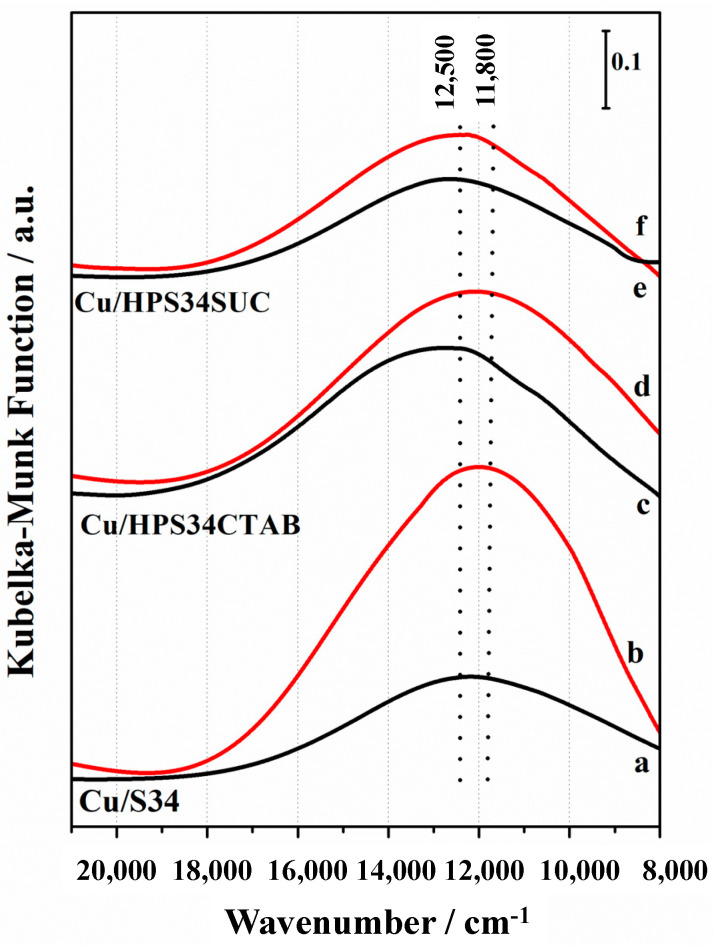
DR UV-Vis spectra in the Vis range of microporous Cu/S34 (a, b curves), hierarchical Cu/HPS34CTAB (c, d curves), and Cu/HPS34SUC (e, f curves) hydrated (black curves) and activated in vacuo at 623 K (red curves).

Copper speciation and coordination in both hierarchical and microporous materials were investigated by diffuse reflectance UV-Vis spectroscopy (DR UV-Vis), focusing on Cu d-d electronic transitions. In fact, in the case of the Cu^2+^, characterized by 3d^9^ electronic configuration, a d-d band will appear in the Vis range, while no features will be visible for colorless Cu^+^ species, characterized by 3d^10^ electronic configuration [7,20]. The DR UV-Vis spectra of hydrated and activated Cu-containing catalysts, reported in Figure 2, revealed the presence of Cu^2+^ d-d bands at 12,500 cm^−1^ for all the hydrated samples; this feature is present in the Vis spectrum of Cu^2+^ ions in aqueous solution and is assigned to distorted octahedral coordination by the Jahn–Teller effect [7,20]. Upon activation of the sample at 623 K in vacuo after calcination, a remarkable shift of the d-d band is observed at 11,800 cm^−1^, but only in the case of the microporous sample (Cu/S34) and of the hierarchical Cu/HPS34CTAB. In contrast, no noticeable shift is visible in the d-d band position for the hierarchical Cu/HP34SUC sample.

The occurrence of this red shift can be attributed to the change in the ligands present within the Cu^2+^ coordination sphere upon thermal activation. This results in the formation of Cu^2+^ sites characterized by square–planar coordination, wherein the adsorbed water molecules acting as ligands in the hydrated forms are effectively replaced by framework oxygen atoms [21].

The evidence of the removal of adsorbed water molecules upon activation is readily observed in the NIR spectra (see Figure S3 in the Supplementary Materials). Specifically, two features in the spectra of the hydrated samples are absent following thermal activation at 623 K. Firstly, the broad signal at 7020 cm^−1^, which arises from the overtone of O-H stretching mode (2ν), becomes undetectable. Additionally, the sharp band located at 5210 cm^−1^, which can be attributed to the combination of stretching and bending modes (ν + δ) of adsorbed water, also vanishes [7,8]. Simultaneously, new signals emerge in the spectra after activation: signals at 4660 and 4550 cm^−1^, due to the combination of stretching and bending modes, and features at 7300 and 7120 cm^−1^, due to overtones of the O-H stretching mode of Brønsted sites, typical of SAPO-34 structure [22], highlighting that not all the OH was exchanged with Cu cations.

**Figure 3:**
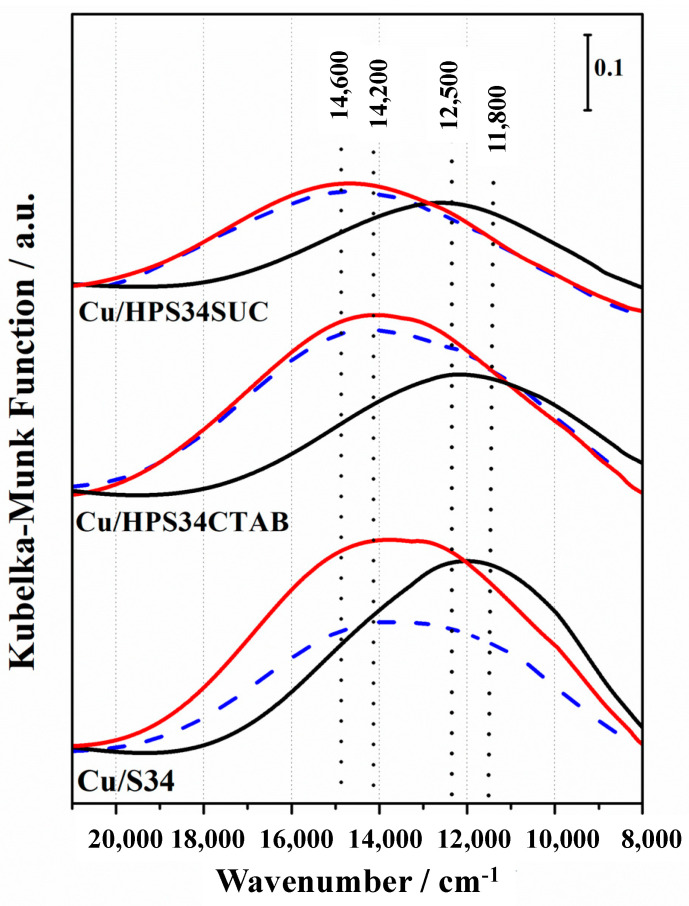
DR UV-Vis spectra in the Vis range of microporous Cu/S34, hierarchical Cu/HPS34CTAB, and Cu/HPS34SUC activated (black curves) upon NH_3_ adsorption (30 mbar, red curves) and upon NH_3_ outgassing at room temperature (blue curves).

On the basis of these features, it is possible to consider that the predominant Cu^2+^ sites formed upon thermal activation in the CHA architecture in microporous and hierarchical Cu/HPS34CTAB catalysts are Z_2_Cu^2+^, where Z is a negative charge delocalized on the framework oxygen atom close to the Al atom, stabilized in the 6 MR. This phenomenon is less evident in hierarchical SAPO-34 prepared with sucrose as a mesoporogen (Cu/HPS34SUC). The accessibility of Cu^2+^ centers was studied by ammonia adsorption at room temperature on activated Cu-containing catalysts (Figure 3). NH_3_ interacts directly with Cu^2+^ sites with the formation of amino complexes and the d-d bands are blue-shifted, indicating the substitution of Cu^2+^ ligands from framework oxygen by NH_3_ molecules [23,24].

Although this blue shift was observed in all samples, it was more evident for the microporous Cu/S34 and the hierarchical Cu/HPS34CTAB. As shown in Figure 3, the band at 11,800 cm^−1^, due to the d-d transition of Cu^2+^ sites in square–planar coordination, is shifted at 14,200 cm^−1^, while the d-d band at 12,500 cm^−1^, due to a more distorted environment, is shifted at 14,600 cm^−1^. The original d-d bands were not restored upon NH_3_ desorption at room temperature (blue curves).

The NH_3_ direct interaction with Brønsted sites can be observed in the NIR region (see Figure S4 in the Supplementary Materials). It can be observed that, upon ammonia adsorption, the bands due to free O–H groups (4660, 4550, 7300, and 7120 cm^−1^) decreased in intensity, and new bands at 6520, 4975 and 4695 cm^−1^ are visible. The signals at 6520 and 4975 cm^−1^ are due to the N–H overtone and stretching and bending combinations, respectively, of ammonia interacting via H-bond with the OH groups of the catalysts; the band at 4695 cm^−1^ can be assigned to the combination of stretching and bending modes of NH_4_^+^ species formed by ammonia protonation by Brønsted sites [25].

**Figure 4:**
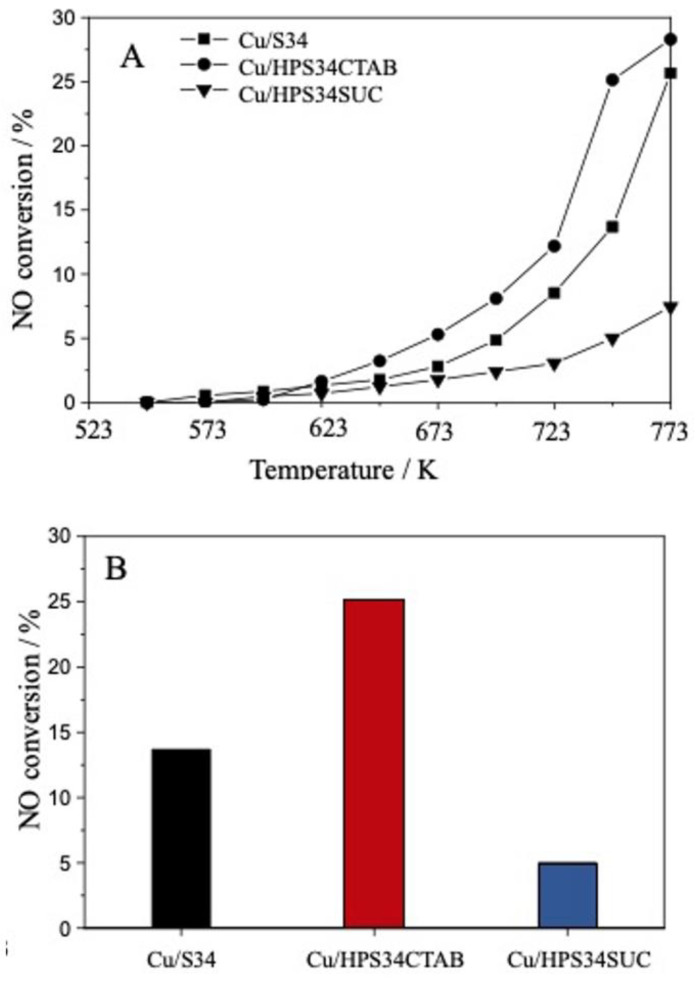
NO conversion for hierarchical and microporous Cu/SAPO-34 with propene as a reductant from 548 K to 773 K (**A**) and sample comparison at 748 K (**B**).

In order to assess the catalytic potential of the hierarchical Cu-based SAPO-34 catalysts, a preliminary evaluation was conducted of the selective catalytic reduction of NO. The reaction was carried out in the presence of either propene or isobutane, which served as the reductants in the system. In stationary powder plants, NH_3_ is usually used as a selective reductant for NO_x_; nevertheless, for automotive applications ammonia has some drawbacks such as NH_3_ slip, storage, and deposit formation [26,27]. For this reason, hydrocarbons can be used as reductants in SCR reactions (HC-SCR). The NO conversion using propene as reductant was performed as a function of temperature from 548 K to 773 K (Figure 4). Overall, Cu/HPS34CTAB showed the highest NO conversion compared with the other Cu-containing SAPO-34. Specifically, at 748 K, Cu/HPS34CTAB had a 25.1% NO conversion, whereas the NO conversions of Cu/S34 and Cu/HPS34SUC were 13.7% and 5.0%, respectively.

The performance of the Cu-containing catalysts was compared by dividing the total amount of NO converted (ppm) at 748 K by the Cu content (µmol), hence giving the amount of NO converted per mol of Cu (Table 3). Interestingly, as Table 3 illustrates, whereas Cu/S34 and Cu/HPS34SUC converted the same amount of NO per mol of Cu present in the sample (30 ppm NO µmol Cu^−1^), Cu/HPS34CTAB had a higher performance value (42 ppm NO µmol Cu^−1^). The best catalytic performances of the hierarchical Cu/HPS34CTAB can be attributed to a synergistic effect between higher Cu loading with respect to the other Cu-containing samples and the hierarchical pore system. Previous reports in fact suggested that the CHA framework can host the Cu sites not only in 6-MR but also, when the Cu loading is >2.4 wt%, inside the larger cages [28,29,30], enhancing the accessibility of Cu sites and the catalytic performances. This phenomenon occurs in hierarchical Cu/HPS34CTAB that has a high percentage of Cu more easily accessible to propene (e.g., in mesopores), thus producing a higher conversion than expected.

**Figure 5:**
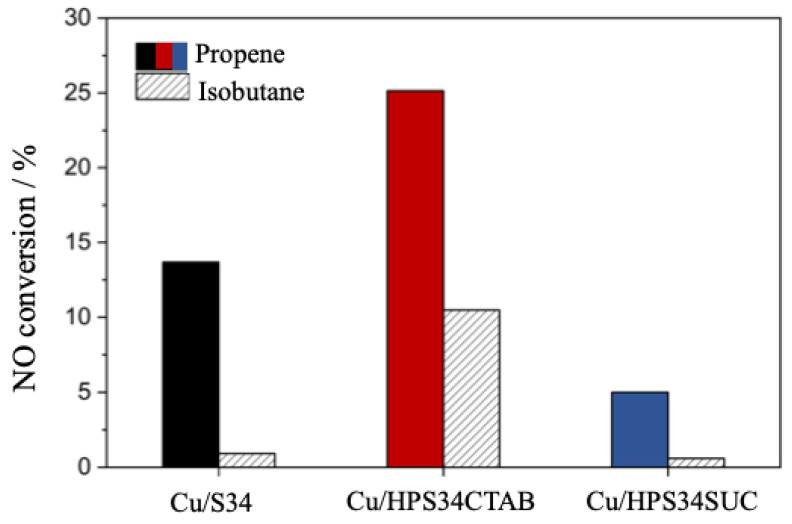
A comparison of the NO conversion using different hydrocarbon reductants (propene and isobutane) at 748 K.

To confirm this hypothesis, HC-SCR was also conducted using isobutane as a reductant (Figure 5). While propene has a kinetic diameter of 0.45 nm and can access the active sites of both the micropores (0.37 nm) and the mesopores (>2 nm) of the CHA framework [31], isobutane has a kinetic diameter of 0.53 nm and can only access active sites in mesopores [32,33]. Thus, by using the inherent shape-selective properties of the CHA framework, the different-sized reductants were used to differentiate the presence of active sites in micropores and/or mesopores [34]. The microporous Cu/S34 had a higher NO conversion when propene was used as a reductant, whereas the NO conversion was very low with isobutane. This is similar to previously reported results on copper-containing conventional SAPO-34 and is because isobutane cannot access the active sites in micropores [32].

The hierarchical Cu/HPS34CTAB, on the other hand, provided NO conversion both when using propene (25%) and when isobutane was used as a reductant (10%), indicating that it has active sites in micropores as well as in mesopores. This also confirms the previously mentioned hypothesis of highly accessible Cu species being the reason for the increased performance of the hierarchical Cu/HPS34CTAB sample when using propene as a reductant. Finally, while 5% of NO conversion was achieved over the hierarchical Cu/HPS34SUC sample using propene, the NO conversion was negligible when using the isobutane reductant (<1%). This indicates that Cu/HPS34SUC has active sites mainly in micropores, i.e., there are few active sites present in the mesopores of this sample.

## 4. Conclusions

Hierarchical SAPO-34 architectures prepared by a bottom-up approach using different structure-directing agents to induce the formation of mesopores, sucrose, and CTAB encapsulated within ordered mesoporous silica nanoparticles were used as hosts for Cu active sites. Cu centers were introduced into the CHA structure by the liquid aqueous solution ion exchange (LIE) method without the SDA removal, avoiding the traditional two-step procedure to prepare Cu-exchanged porous materials.

The physicochemical characterization evidenced the different effect of the mesoporogenic templates; the hierarchical HPS34CTAB had a higher mesopore surface area and volume with respect to the hierarchical SAPO-34 prepared using sucrose, highlighting the role of the hierarchical architecture in the exchange capacity of the materials. The HPS34CTAB, in fact, was able to host a high Cu loading compared with the other Cu-exchanged samples, and the Cu-exchanged sites showed a coordination and behavior similar to that of the microporous counterpart.

The improved performances of the Cu/HPS34CTAB catalyst in the selective catalytic reduction with hydrocarbons of NO can be attributed to the synergistic effects resulting from multiple factors. Firstly, the hierarchical pore architecture of the CHA structure played a vital role in facilitating enhanced catalytic activity. Additionally, the high loading of Cu within the catalyst, combined with the precise location and accessibility of the active sites, further contributed to its better performance.

In addition, the results of the catalytic tests using propene or isobutane as reductants in the selective catalytic reduction provide valuable insights. It was observed that the Cu/HPS34CTAB exhibited a higher fraction of Cu ions allocated within highly accessible mesopores. This allocation pattern leads to improved activity in terms of NO conversion when compared to the other Cu-based SAPO-34 catalysts.

## Figures and Tables

**Table 1:** Textural properties of hierarchical and microporous SAPO-34 catalysts.

Materials	S_BET_ (m^2^/g)	S_DFT_ (m^2^/g)	S_micro_ (m^2^/g)	^a^ S_meso_ (m^2^/g)	V_tot DFT_ (cm^3^/g)	V_micro_ (cm^3^/g)	V_meso_ (cm^3^/g)
HPS34CTAB	641	783	511	272	0.58	0.13	0.44
HPS34SUC	554	808	590	118	0.36	0.17	0.19
S34	477	819	811	8	0.26	0.23	0.03

^a^ S_meso_ = S_DFT_ − S_micro_.

**Table 2:** Acronyms, framework composition, structure-directing agents (SDAs), and Cu loadings calculated from UV-Vis spectroscopy.

Acronyms	Framework Elements			SDA		Cu Loading/wt%
	Al	Si	P	SDA_meso_	SDA_micro_	
Cu/HPS34CTAB	1	0.6	1	0.067	1	2.56
Cu/HPS34SUC	1	0.6	1	0.067	1	0.70
Cu/S34	1	0.6	1	-	1	1.92

**Table 3:** NO conversion and NO conversion per weight percent of Cu for all samples.

Catalysts	NO Conversion (%)	Cu Loading (wt.%)	NO Converted per µmol Cu (ppm µmol^−1^)
Cu/HPS34CTAB	25.1	2.56	42
Cu/HPS34SUC	5	0.7	30
Cu/S34	13.7	1.92	30

## Data Availability

Not applicable.

## Supplementary Materials

The following supporting information can be downloaded at: https://www.mdpi.com/article/10.3390/ma16165694/s1.
