# Supplementary material for: Hierarchical SAPO-34 Catalysts as Host for Cu Active Sites
Source: Materials (Basel). 2023 Aug 19;16(16):5694. doi: 10.3390/ma16165694 (PMC10456513; doi:10.3390/ma16165694)
Supplement: Supplementary file 1 [file materials-16-05694-s001.zip › materials-2544441-supplementary.pdf]

Supporting Information

Hierarchical SAPO-34 Catalysts as Host for Cu Active Sites

Julio C. Fernandes Pape Brito [1], Ivana Miletto [2], Leonardo Marchese [3], Daniel Ali [4], Muhammad Mohsin Azim [4], Karina Mathisen [4] and Enrica Gianotti [1,*]

[1] Department for Sustainable Development and Ecological Transition, Università del Piemonte Orientale, Piazza Sant'Eusebio 5, 13100 Vercelli, Italy
[2] Department of Pharmaceutical Sciences, Università del Piemonte Orientale, Largo Donegani 2, 28100 Novara, Italy
[3] Department of Science and Technological Innovation, Università del Piemonte Orientale, Via T. Michel 11, 15100 Alessandria, Italy; leonardo.marchese@uniupo.it
[4] Department of Chemistry, Norwegian University of Science and Technology (NTNU), 7491 Trondheim, Norway
* Correspondence: enrica.gianotti@uniupo.it

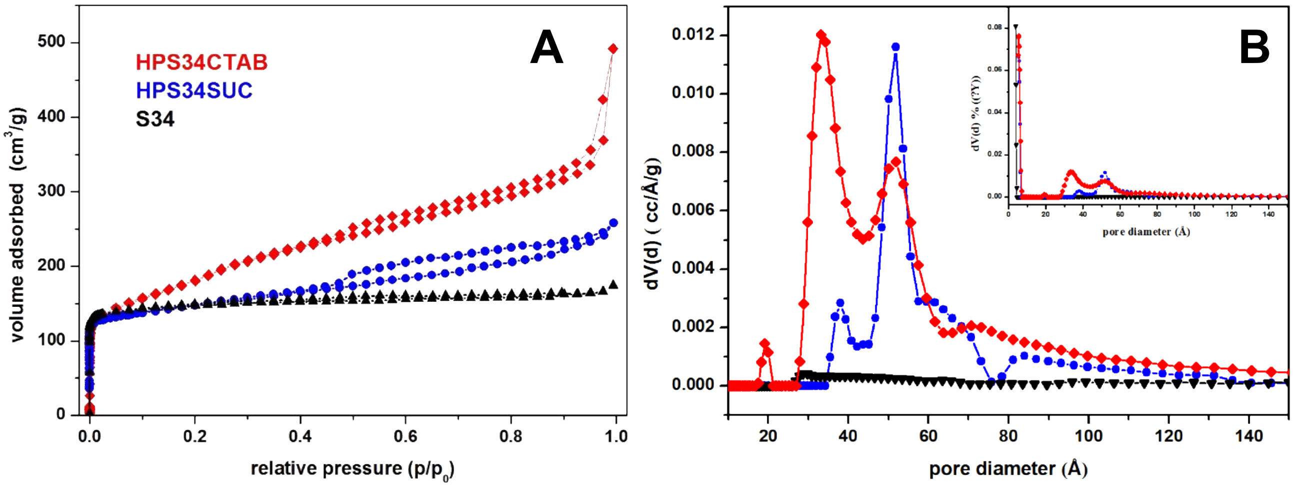

Fig. S1 - N_2 adsorption/desorption isotherms at 77K (section A) and pore size distribution (section B) of HPS34CTAB (red diamonds), HPS34SUC (blue circles) and S34 (black triangles)

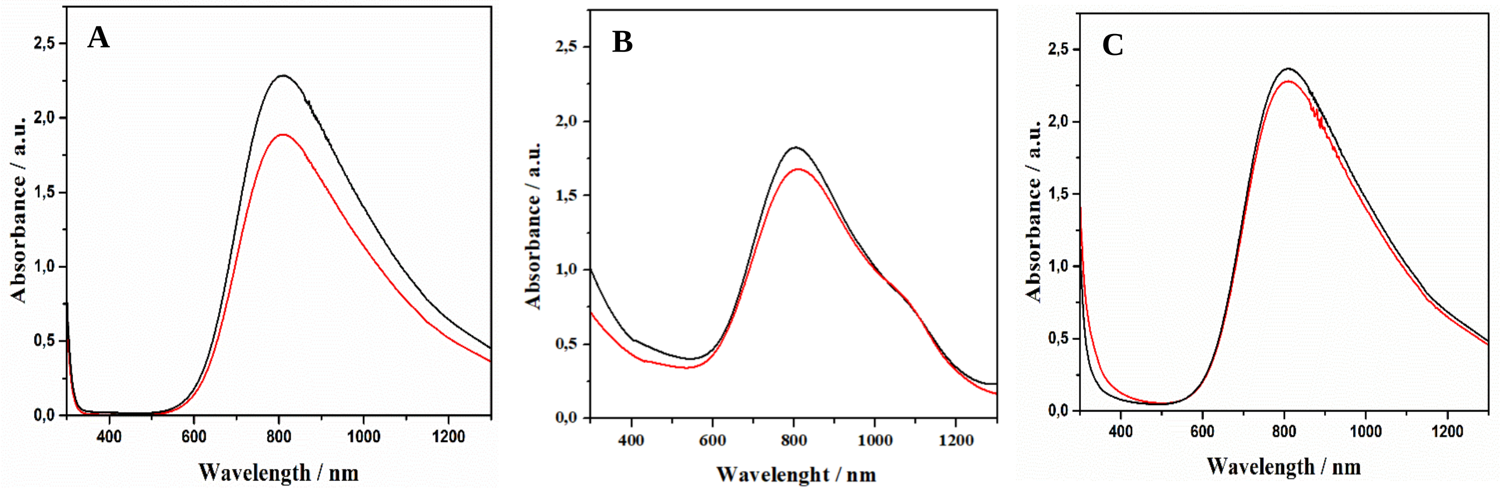

Fig.S2- UV-Vis spectra of eluate after the first (red curve) and the second (black curve) washing procedure. Eluated water of S34 (Section A), HPS34CTAB (Section B) and HPS34SUC (Section C).

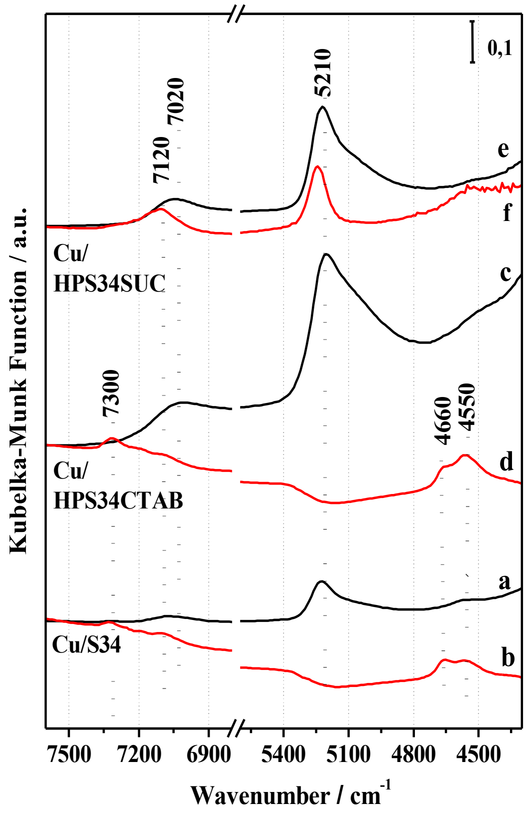

Fig.S3: DR NIR spectra of microporous Cu/S34 (a, b curves), hierarchical Cu/HPS34CTAB (c, d curves) and Cu/HPS34SUC (e, f curves) hydrated (black curves) and activated at 623 K (red curves).

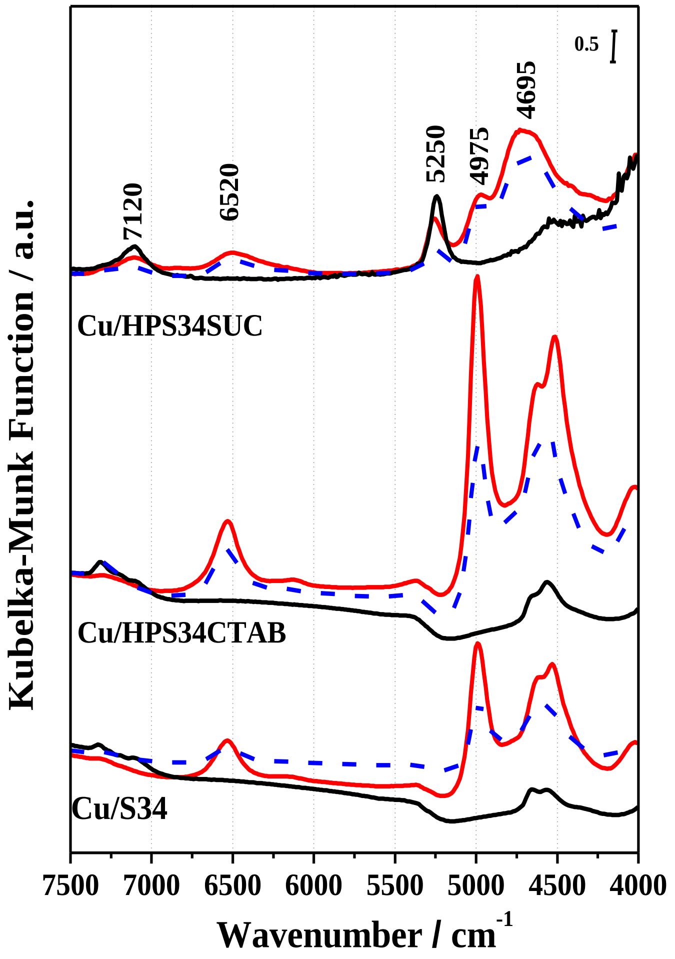

Fig S4. DR NIR spectra of microporous Cu/S34, hierarchical Cu/HPS34CTAB and Cu/HPS34SUC activated (black curves), upon NH_3 adsorption (30 mbar, red curves) and upon NH_3 outgassing at room temperature (blue curves).
